# Diagnostic Performance of Inflammatory Biomarkers in Pediatric Acute Appendicitis

**DOI:** 10.3390/children13060838

**Published:** 2026-06-22

**Authors:** Hilmi Onur Kabukçu, Sarper Müftüoğulları, Eren Yıldız

**Affiliations:** 1 Department of Pediatrics, Private Kastamonu Anatolia Hospital, Kastamonu 37200, Turkey; onurkabukcu89@gmail.com; 2 Department of Pediatric Surgery, Kastamonu Training and Research Hospital, Kastamonu 37150, Turkey; sarpermft@gmail.com; 3 Department of Pediatrics, Faculty of Medicine, Kastamonu University, Kastamonu 37150, Turkey

**Keywords:** pediatric acute appendicitis, fibrinogen, biomarker, complicated appendicitis

## Abstract

**Objectives:** The diagnosis of pediatric acute appendicitis remains challenging due to clinical findings that overlap with nonspecific abdominal pain (NSAP). In this study, the value of fibrinogen in the diagnosis of pediatric acute appendicitis and in the classification of disease severity was investigated. **Methods:** In a single-center, retrospective cohort study, 145 patients aged 1 month to 18 years who underwent contrast-enhanced abdominal computed tomography were divided into three groups: NSAP (n = 62), uncomplicated appendicitis (n = 44), and complicated appendicitis (n = 39). Hemogram parameters, CRP, procalcitonin, albumin, and fibrinogen levels were compared. Diagnostic performance was assessed using ROC analysis, and independent predictors were evaluated via multivariate logistic regression. **Results:** Fibrinogen levels showed a gradual and statistically significant increase from NSAP to uncomplicated appendicitis and then to complicated appendicitis (*p* < 0.001 for all pairwise comparisons). In distinguishing appendicitis from NSAP, fibrinogen achieved the highest diagnostic accuracy among the biomarkers examined (AUC = 0.95); CRP, WBC, ANC, and NLR demonstrated lower discriminatory performance. In multivariate logistic regression analysis, fibrinogen was validated as an independent predictor of appendicitis (*p* < 0.001). **Conclusions:** Fibrinogen demonstrates high discriminatory performance in the diagnosis of pediatric acute appendicitis and shows a graded relationship with disease severity. These findings suggest that fibrinogen may be a promising biomarker for the evaluation of pediatric acute appendicitis. However, larger prospective multicenter studies are required before its routine integration into diagnostic algorithms can be recommended.

## 1. Introduction

Acute appendicitis is the most common surgical emergency encountered in pediatric emergency departments, and its diagnosis is based on a combination of clinical assessment, repeated physical examinations, scoring systems, imaging modalities, and laboratory biomarkers [1]. In spite of the development of diagnostic techniques, complicated forms of acute appendicitis, such as perforation, peritonitis, and gangrenous appendicitis, occur in 30% of cases where acute appendicitis is identified, especially in infants below five years. Early identification of complicated forms of acute appendicitis is crucial for their impact on the length of hospital stay, mortality, and morbidity [2,3]. The ability of the Pediatric Appendicitis Score (PAS) to identify complicated appendicitis is limited, as misdiagnosis rates may reach up to 30%, and the score does not reliably distinguish complicated from uncomplicated appendicitis. It is assumed that this issue emerges as a result of frequent atypical symptoms observed in about 50% of patients, and also difficulties in taking patient history, especially among infants under five years old [4].

Although imaging tests help in diagnosing the condition, there are many drawbacks associated with them. Ultrasonography is a rapid and noninvasive imaging modality; however, it remains highly operator-dependent, and visualization of the appendix may be challenging because of factors such as patient body habitus, bowel gas, operator experience, and variable appendix location, including retrocecal appendicitis [5]. Because of this, physicians prefer to go for computed tomography (CT), which, unfortunately, poses some risks related to radiation exposure, contrast nephropathy, and anaphylaxis in children [6]. Therefore, there is an increasing requirement for biochemical markers apart from imaging techniques. Among the most frequently used markers are blood tests, CRP, procalcitonin, albumin, and sodium; however, it has been established that an elevation of these markers may occur in various diseases leading to abdominal pain, including acute gastroenteritis, with relatively low efficiency of their use in diagnosing complicated and uncomplicated appendicitis [2,3,7].

As a way to counteract this problem, fibrinogen—an acute phase reactant produced through interleukin-6—has been getting more attention lately. Research conducted among adults has proven its efficiency in both differential diagnostics and complicated appendicitis diagnostics [8,9]; nevertheless, there is very little research conducted among pediatrics concerning this subject [10]. The main aim of this study is to compare the ability of hematological markers (fibrinogen being the primary one) and CRP, procalcitonin, albumin, sodium, AST, and ALT levels to diagnose acute appendicitis and complicated and uncomplicated appendicitis, respectively. The secondary objective of this research paper is to assist in reducing the incidence of unnecessary surgeries by establishing the cut-off levels of key markers.

## 2. Materials and Methods

This study includes 145 pediatric cases aged 1 month to 18 years who presented to a tertiary pediatric emergency department with complaints of acute abdominal pain and underwent contrast-enhanced abdominal CT between September 2024 and July 2025. At the time of presentation, patients were evaluated by a single pediatric surgeon and classified as NSAP or clinical acute appendicitis based solely on clinical evaluation and abdominal CT imaging.

### 2.1. Data Collection

Contrast-enhanced abdominal computed tomography was performed for all cases at the time of presentation, and concurrently, complete blood count and biochemical parameters were analyzed from a single venous blood sample in accordance with the hospital’s routine laboratory procedures. From the complete blood count, white blood cell (WBC) count, absolute neutrophil count (ANC), absolute lymphocyte count (ALC), neutrophil-to-lymphocyte ratio (NLR), monocyte count, monocyte-to-lymphocyte ratio (MLR), platelet (PLT) count, and red blood cell distribution width (RDW) were calculated. RDW/PLT ratio and PLT/WBC ratio were calculated. Biochemical parameters included CRP, procalcitonin, AST, ALT, sodium, fibrinogen, and albumin levels. Regarding sociodemographic data, age and gender information for all cases were recorded.

Patients diagnosed clinically with acute appendicitis via CT underwent laparoscopic or open appendectomy under emergency conditions. The diagnosis was confirmed by intraoperative findings and histopathological examination. Patients with a confirmed diagnosis of acute appendicitis were classified as having uncomplicated or complicated appendicitis according to histopathological findings and operative records. Complicated appendicitis was defined by the presence of perforation, gangrene, or peritonitis. Cases of complicated appendicitis were further categorized as perforated, gangrenous, or peritonitic appendicitis for subgroup analyses. The NSAP group consisted of cases in which acute appendicitis was not suspected based on clinical evaluation and abdominal CT, as well as cases in which appendicitis was not detected following appendectomy. Cases in which acute appendicitis was not suspected were followed up for 2 weeks to rule out the diagnosis.

### 2.2. Exclusion Criteria

The exclusion criteria are as follows:

1. Chronic inflammatory disease (e.g., Crohn’s disease, ulcerative colitis)
2. Patients with a history of immunodeficiency or malignancy
3. Patients who have had a systemic infection within the past month
4. Individuals currently receiving corticosteroid or immunomodulatory therapy
5. Individuals with missing laboratory data or follow-up records
6. Patients who have previously undergone appendectomy
7. Patients with abdominal pain due to trauma
8. Patients with familial Mediterranean fever and celiac disease

### 2.3. Statistical Analysis

Statistical analyses were performed using SPSS version 25.0 (IBM Corp., Armonk, NY, USA). The Kolmogorov- Smirnov test was used to assess the normality of distribution. Descriptive statistics were presented as frequency, percentage, mean, standard deviation (SD), median, and the 25th (Q1) and 75th (Q3) quartiles. When evaluating continuous variables between two independent groups, Student’s *t*-test and the Mann–Whitney U test were used depending on the normality of the distribution; when comparing three groups, one-way ANOVA and the Kruskal–Wallis test were applied. Bonferroni correction was applied in post hoc multiple comparisons, and corrected *p*-values were presented. The chi-square test was used to compare categorical variables. ROC (receiver operating characteristic) analysis was performed to compare the predictive power of indices that could be used in the diagnosis of appendicitis. To ensure a more accurate assessment of diagnostic performance, only variables showing significant differences between groups were included in the ROC analysis. Thus, the sensitivity, specificity, and cut-off values of parameters with potential clinical discriminatory value were calculated. The area under the curve (AUC) was interpreted as follows: <0.5: useless, 0.5–0.7: poor, 0.7–0.9: good, 0.9–1.0: excellent. Additionally, binary logistic regression analysis was performed to identify independent factors associated with the diagnosis of appendicitis. Variables showing statistical significance in the univariate analyses and those considered clinically relevant were included in the multivariate model. Prior to model construction, multicollinearity among candidate variables was assessed using the variance inflation factor (VIF) and tolerance statistics. Variables demonstrating substantial multicollinearity were excluded from the final model. Adjusted odds ratios (ORs) with 95% confidence intervals (CIs) were calculated for all variables included in the logistic regression analysis. Model fit was evaluated using the Hosmer–Lemeshow goodness-of-fit test. A *p*-value of <0.05 was considered statistically significant.

### 2.4. Ethics Committee Approval

This study was approved by the Kastamonu University Ethics Committee for Non-Interventional Clinical Research (Approval No.: 2025-26; Date: 16 October 2025). All procedures were conducted in accordance with the principles of the Declaration of Helsinki.

## 3. Results

### 3.1. Study Population

A total of 145 pediatric patients were included in the study, comprising 62 (42.7%) patients in the NSAP group, 44 (30.3%) patients in the uncomplicated appendicitis group, and 39 (26.9%) patients in the complicated appendicitis group. There was a statistically significant difference in gender distribution within each group (*p* < 0.001), but males within the complicated appendicitis group comprised a significantly greater proportion of the study population than did males within the other two groups. However, no difference existed in the distribution of age in any of the three groups (*p* = 0.930) (Table 1).

### 3.2. Comparison of Laboratory Parameters Among the Three Groups

Comparison of laboratory parameters across the three groups demonstrated a progressive increase in inflammatory markers with increasing disease severity. WBC (*p* = 0.008), ANC (*p* = 0.002), NLR (*p* = 0.003), monocytes (*p* = 0.008), and MLR (*p* = 0.006) were significantly elevated only in the complicated appendicitis group, while CRP and fibrinogen were significantly elevated both in the uncomplicated and complicated appendicitis groups in comparison with the NSAP group (*p* < 0.001). Fibrinogen was detected to be 319.5, 435.0, and 526.0 mg/dL (median) in the NSAP, uncomplicated appendicitis, and complicated appendicitis groups, respectively, and was statistically significant in the comparison among all the groups (*p* < 0.001). On the other hand, the PLT/WBC ratio was significantly reduced only in the complicated appendicitis group (*p* = 0.014), while the serum sodium level was significantly decreased in the complicated appendicitis group (*p* = 0.008), and the albumin level was significantly higher in the NSAP group (*p* < 0.001). Detailed comparisons are presented in Table 1.

### 3.3. Comparison of Laboratory Parameters Between the NSAP and Appendicitis Groups

The statistical analysis showed that when the parameters of the appendicitis group were evaluated in comparison with the NSAP group, WBC (*p* = 0.033), ANC (*p* = 0.012), NLR (*p* = 0.028), and monocyte (*p* = 0.015) values were significantly higher in the appendicitis group. The median value of CRP (*p* < 0.001) and fibrinogen (*p* < 0.001) levels were significantly higher in the appendicitis group. In the NSAP group, median fibrinogen levels were 319.5 mg/dL, while in the appendicitis group, they were 491.0 mg/dL. PLT/WBC ratio (*p* = 0.015) and albumin (*p* < 0.001) were significantly lower in the appendicitis group. There were no statistically significant differences between other parameters (Table 2).

### 3.4. Comparison of Subgroups of Complicated Appendicitis

Complicated appendicitis patients were evaluated in relation to three different categories: perforated (n = 15), gangrenous (n = 14), and peritonitis (n = 10). While there was a higher incidence of male subjects in all three categories when considering their gender, this was not a statistically significant finding (*p* = 0.302). There was also no statistically significant difference found when their age distribution was considered (*p* = 0.528). In terms of laboratory findings, there were no significant differences for all parameters measured (Table 3).

### 3.5. Diagnostic Performance of Laboratory Parameters

**Figure 1:**
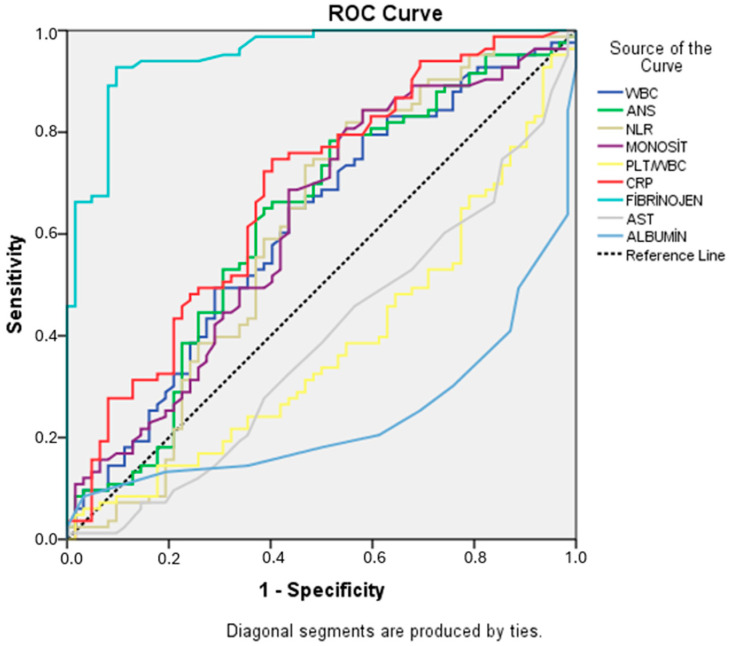
Receiver operating characteristic (ROC) curve analysis and AUC values.

A ROC curve was generated to determine the discriminatory power of biochemical parameters for appendicitis compared to NSAP. It was found that fibrinogen had the highest AUC value (AUC = 0.95; 95% CI: 0.92–0.98), and at the cut-off point of 395.0 mg/dL, the sensitivity and specificity reached 92.8% and 90.3%, respectively. CRP (AUC = 0.68), WBC (AUC = 0.61), ANC (AUC = 0.62), NLR (AUC = 0.61), and monocytes (AUC = 0.62) exhibited lower AUC values. The AUC values for other parameters are presented in Table 4 (Table 4, Figure 1).

### 3.6. Multivariate Logistic Regression Analysis

A multivariate logistic regression analysis was performed to identify independent risk factors associated with the presence of appendicitis. Prior to model construction, multicollinearity among candidate variables was assessed using the variance inflation factor (VIF) and tolerance statistics. Because significant multicollinearity was detected between WBC and ANC, ANC was excluded from the final multivariate model. The overall logistic regression model was statistically significant (Omnibus test: χ^2^ = 143.492, *p* < 0.001) and demonstrated excellent explanatory power (Nagelkerke R^2^ = 0.844). The Hosmer–Lemeshow goodness-of-fit test indicated adequate model calibration (χ^2^ = 11.010, *p* = 0.201). The model correctly classified 93.1% of the study participants.

In the final multivariate logistic regression model, fibrinogen (OR: 1.048; 95% CI: 1.028–1.068; *p* < 0.001), albumin (OR: 0.044; 95% CI: 0.005–0.379; *p* = 0.004), CRP (OR: 0.969; 95% CI: 0.945–0.994; *p* = 0.016), and AST (OR: 0.921; 95% CI: 0.849–0.998; *p* = 0.045) were identified as independent predictors of appendicitis. Other variables, including sex, WBC, NLR, monocyte count, and PLT/WBC ratio, were not found to be independently associated with appendicitis (Table 5).

## 4. Discussion

In this study, it was found that the inflammatory markers examined in pediatric cases with acute appendicitis showed a parallel increase with disease severity. As for increases in WBC, ANC, NLR, and monocytes, these were exclusively seen in the complicated appendicitis group; CRP and fibrinogen, on the other hand, showed significant differences in both the uncomplicated and complicated appendicitis groups, as compared to the NSAP group. Notably, fibrinogen levels increased progressively from the NSAP group to the uncomplicated appendicitis group and further to the complicated appendicitis group, with statistically significant differences observed between all groups. It is also notable that the PLT/WBC ratio and albumin were decreased in the appendicitis group as compared to the NSAP group. In terms of diagnostic performance, fibrinogen demonstrated the highest discriminatory performance among all parameters examined. Furthermore, in multivariate logistic regression analysis, fibrinogen and albumin were identified as independent predictors of appendicitis.

Acute appendicitis is the most common surgical pathology encountered in emergency departments in children, just as it is in adults [10]. However, recognizing the differences between acute appendicitis and NSAP and making the necessary distinctions between complicated and uncomplicated appendicitis is essential clinically since it prevents unnecessary surgeries and complications. Although imaging modalities are helpful in diagnosing cases, the operator dependence of ultrasound and radiation, nephrotoxicity, and anaphylactic reactions of CT necessitate their cautious use [1,6]. Especially in children, the demand for minimally invasive and readily available diagnostic options other than or beside imaging, considering the long-term risk of cancer caused by ionizing radiation, is increasing. In recent years, extensive research has focused on the diagnostic utility of inflammatory and coagulation parameters. In particular, interest in inflammatory biomarkers such as fibrinogen in pediatric acute appendicitis has increased substantially. Furthermore, a systematic review and meta-analysis by Wu et al. involving 3136 patients demonstrated that fibrinogen showed superior diagnostic performance for complicated appendicitis, with a pooled sensitivity of 74%, specificity of 76%, and an AUC of 0.84 [11]. Therefore, as observed in our study, the interest in diagnostic biomarkers useful for identifying appendicitis from NSAP and complicated appendicitis from uncomplicated appendicitis is increasing in the current literature.

In our study, when the appendicitis and NSAP groups were compared, the appendicitis group was found to have significantly higher levels of hemogram parameters such as WBC, ANC, NLR, and monocytes, as well as CRP and fibrinogen, while albumin levels were lower. Nevertheless, despite the evident elevation of the WBC, ANC, and NLR values in the group of complicated appendicitis only, CRP and fibrinogen turned out to increase in both the complicated and uncomplicated appendicitis groups. It is important to note that only fibrinogen showed a steady increase depending on the increasing inflammation level caused by the condition. An analysis of pediatric studies found in the literature shows that one 2021 meta-analysis conducted on 5974 patients from 19 trials found that the best single biomarker for diagnosing appendicitis as opposed to non-acute abdominal pain was NLR; however, it is not a sufficient marker itself [12]. One recent multicenter retrospective study conducted by Ding et al. [13] involved 323 pediatric patients and found that, after using a selection method based on LASSO algorithms, independent predictors such as neutrophils, CRP, fibrinogen, and chloride could be identified as independent predictors of complicated appendicitis, with the nomogram model developed using these predictors being more accurate than the Alvarado and PASs [13]. Upon reviewing international guidelines, the WSES 2025 [14], SAGES 2024 [15], and EAES 2015 [16] recommend the combination of WBC and CRP for the differential diagnosis of acute appendicitis; however, they emphasize that these parameters are insufficient for establishing a diagnosis on their own and can only play a supplementary role in conjunction with imaging methods. When studies on the distinction between complicated and uncomplicated appendicitis are evaluated, it is noteworthy that research focusing on CRP and fibrinogen has received increasing attention compared with studies evaluating conventional hemogram parameters [7,8,9,10,17]. Furthermore, a systematic review and meta-analysis by Wu et al. involving 3136 patients demonstrated that fibrinogen showed superior diagnostic performance for complicated appendicitis, with a pooled sensitivity of 74%, specificity of 76%, and an AUC of 0.84 [11]. The results of these studies are largely consistent with our findings. Considering the pathophysiology of acute appendicitis, an inflammatory response is activated secondary to lumen obstruction and bacterial proliferation; the elevation of inflammatory markers in the systemic circulation during this process explains the increases in biomarkers observed in our study [1].

WBC, ANC, NLR, and CRP, among other routine biochemical markers, are included in the diagnosis of acute appendicitis guidelines and are commonly applied in medical practice. Nevertheless, they are prone to several weaknesses. While CRP serves as an excellent marker of inflammation, its late appearance makes it inappropriate for diagnosing appendicitis [17]. Several researchers have pointed out that hematology markers like WBC, ANC, and NLR are characterized by high sensitivity but low specificity and fail to adequately differentiate complicated from uncomplicated appendicitis [2,3,5,7]. In a study conducted with 90 pediatric patients, Narsat et al. indicated that there was no statistical difference between complicated and uncomplicated appendicitis as far as WBC and absolute neutrophil count were concerned, although CRP was identified as an independent predictor in multivariate analysis [18]. These findings suggest that conventional biomarkers may be insufficient when used alone and are likely to provide greater value when interpreted in combination with other diagnostic methods. research focusing on CRP and fibrinogen rather than hemogram parameters takes center stage. Fibrinogen, which is an acute-phase protein formed by hepatic cells through interleukin-6, has been found to be very useful in many studies lately in diagnosing acute appendicitis and predicting its complications. Recent studies have highlighted the role of IL-6 as a promising biomarker in pediatric appendicitis. Elevated IL-6 concentrations have been associated with both the diagnosis and severity of appendicitis, supporting the concept that activation of the IL-6-mediated inflammatory pathway contributes substantially to disease progression. Given that fibrinogen is synthesized in response to IL-6 stimulation, the strong performance of fibrinogen observed in our study may partially reflect this underlying biological mechanism [8,12,17]. In terms of mechanisms, the higher synthesis of fibrinogen induced by IL-6 in the inflammatory response, the activation of the extrinsic coagulation pathway through tissue factor, and the occurrence of hypercoagulability in accordance with the extent of tissue injury account for the sensitivity of this parameter in assessing the severity of the inflammatory process compared to other parameters [11,13]. In our study, fibrinogen levels increased in parallel with appendicitis severity, suggesting that this parameter may more accurately reflect the intensity of the inflammatory process. Therefore, fibrinogen may serve as a useful complementary biomarker for the diagnosis of appendicitis and the assessment of disease severity.

When considering the diagnostic ability of the parameters analyzed in this study, ROC analysis indicated that fibrinogen was the best diagnostic marker of all. As such, the AUC value for fibrinogen of 0.95 indicated high diagnostic power in differentiating between appendicitis and NSAP, with the sensitivity and specificity of 92.8% and 90.3%, respectively, at a cut-off value of approximately 395 mg/dL. When reviewing literature for other studies on this topic, it becomes apparent that cut-off values reported by other researchers vary greatly. Specifically, in a cross-sectional study involving 82 patients in different age categories, Nyuwi et al. found 82% sensitivity and 60% specificity at a cut-off value of 397 mg/dL, and when reduced to 375 mg/dL, a sensitivity of 88% and specificity of 55% were observed, with AUC equal to 0.697 [19]. In a cohort study by Shafagh et al. [20], on the adult population, the sensitivity of this test is 66.7%, the specificity is 92.8%, while the AUC is 0.892 at the cut-off point of 272 mg/dL. In addition, it was mentioned that fibrinogen can be considered a complementary biomarker, particularly in cases where there is disagreement between clinical information and results from CRP [20]. There may have been considerable variation in the cut-off values because of variations in the age group of the sample population, the length of the duration of the disease, measuring fibrinogen, and the definition of the diseases NSKA and complicated appendicitis. The value of AUC obtained in our study (0.95) is significantly greater than the pooled data presented in the literature (0.77 for simple appendicitis and 0.84 for complicated appendicitis according to the meta-analysis by Wu et al.) [11]. It should be noted that such a difference may be explained by selection bias as well as a small number of participants included in the study. The exceptionally high diagnostic performance of fibrinogen observed in our study should be interpreted with caution. Compared with previous studies and meta-analytic estimates, our cohort was relatively small and consisted exclusively of patients who underwent CT evaluation, potentially enriching the population for diagnostically distinct cases. These factors may have increased the apparent discriminatory performance of fibrinogen. Therefore, although our findings support the potential value of fibrinogen, external validation in larger prospective multicenter cohorts is required before routine clinical implementation can be recommended. As far as the performance of other biomarkers, CRP, WBC, ANC, NLR, and monocytes have been shown to perform quite poorly in terms of their diagnostic power. Taken together, our findings suggest that fibrinogen may have promising diagnostic value and may contribute to clinical decision-making when interpreted alongside clinical findings and other laboratory parameters. The results of the multivariate logistic regression analysis did not prove conventional markers to be independent risk factors, but only fibrinogen (OR = 1.050, 95% CI = 1.028–1.073; *p* < 0.001) was identified as an independent predictor, which is consistent with the ROC analysis findings. The results of this study suggest that elevated fibrinogen levels are associated with appendicitis and may reflect the intensity of the underlying inflammatory process. Nonetheless, the possibility that the diagnostic and predictive accuracy of the biomarker may not be established in other populations should not be ignored, and the results should be interpreted within this framework. Consequently, a multicenter and prospective study is needed in order to standardize the use of fibrinogen as a marker for pediatric acute appendicitis.

The ability to distinguish uncomplicated from complicated appendicitis may have important clinical implications beyond diagnosis alone. Early identification of patients at increased risk for complicated appendicitis may facilitate surgical planning, optimize perioperative management, and support timely involvement of experienced surgical teams when necessary. In this regard, the progressive increase in fibrinogen levels observed with increasing disease severity suggests that fibrinogen may provide additional information for risk stratification in children with acute appendicitis.

There are some limitations associated with this study. First, the retrospective design of the study does not allow causal relationships to be established. Second, being based on the data from a single center, the results may not be generalized to other populations. Third, all laboratory markers were determined during a single hospital visit when the patients presented symptoms; hence, no information about the time that elapsed between the development of symptoms and testing was provided, and the time-dependent changes in fibrinogen levels throughout the disease course could not be examined. Fourth, there was no evaluation of the effectiveness of using various combinations of the tested markers. Fifth, only patients who underwent contrast-enhanced abdominal CT were included in the study. Because many children with suspected appendicitis are diagnosed using clinical assessment and ultrasonography without CT imaging, our cohort may represent a more diagnostically challenging subgroup. This selection criterion may have introduced selection bias and may limit the generalizability of the findings to the broader pediatric appendicitis population. Additionally, the interval between symptom onset and laboratory testing was not available because of the retrospective design of the study. Therefore, temporal changes in fibrinogen and other inflammatory biomarkers throughout the disease course could not be evaluated. Finally, established clinical scoring systems such as the Pediatric Appendicitis Score (PAS), Alvarado score, and Appendicitis Inflammatory Response (AIR) score were not evaluated. Therefore, the incremental diagnostic value of fibrinogen beyond these validated clinical tools could not be assessed. Thus, there is a need for multicenter and prospective research.

Overall, fibrinogen demonstrated promising diagnostic performance in pediatric acute appendicitis and showed a graded association with disease severity. Compared with traditional inflammatory markers, fibrinogen may provide additional diagnostic information for distinguishing appendicitis from nonspecific abdominal pain and for identifying patients at risk of complicated disease. However, given the retrospective single-center design and the potential for selection bias, these findings should be interpreted with caution. Larger prospective multicenter studies are required to externally validate our results before routine clinical implementation can be recommended.

## Figures and Tables

**Table 1:** Comparison of demographic and biochemical parameters across groups.

Variable	Healthy Controls (n = 62)		Uncomplicated Appendicitis (n = 44)		Complicated Appendicitis (n = 39)		*p*
**Sex**							
**Female, n (%)**	36 (58.1)		23 (52.3)		7 (17.9)		<0.001 *
**Male, n (%)**	26 (41.9)		21 (47.7)		32 (82.1)		
	**Mean ± SD**	**Median** **(Q1–Q3)**	**Mean ± SD**	**Median** **(Q1–Q3)**	**Mean ± SD**	**Median** **(Q1–Q3)**	
**Age (month)**	156.4 ± 41.9	165.5 (126.0–189.0)	153.7 ± 46.7	153.5 (111.5–200.0)	153.7 ± 44.1	161.0 (127.5–190.0)	0.930 ^K^
**WBC**	12.1 ± 4.6	11.2 (8.6–14.9)	12.7 ± 5.1	12.0 (9.9–14.7)	15.1 ± 4.9	14.9 (11.4–17.7)	**0.008 ^A^** **a–c = 0.007 ^M^**
**ANC**	8.8 ± 4.7	7.8 (5.0–11.6)	9.6 ± 5.1	9.0 (6.0–11.4)	12.0 ± 4.7	12.3 (8.6–14.4)	**0.002 ^K^** **a–c = 0.004 ^M^**
**ALS**	2.3 ± 1.4	2.2 (1.2–3.0)	2.2 ± 1.0	2.1 (1.3–2.6)	1.8 ± 0.9	1.8 (1.3–2.3)	0.118 ^A^
**NLR**	6.3 ± 6.4	3.5 (1.9–7.3)	5.4 ± 3.8	4.2 (2.8–7.3)	8.1 ± 5.8	7.1 (4.5–9.7)	**0.003** **^K^** **a–c = 0.003 ^M^** **b–c = 0.004 ^M^**
**Monocyte**	0.8 ± 0.4	0.7 (0.5–1.1)	0.9 ± 0.3	0.8 (0.6–1.0)	1.0 ± 0.4	0.9 (0.8–1.3)	**0.008** **^K^** **a–c = 0.004 ^M^**
**MLR**	0.5 ± 0.3	0.4 (0.2–0.6)	0.5 ± 0.2	0.4 (0.3–0.6)	0.7 ± 0.5	0.5 (0.4–0.9)	**0.006** **^K^** **a–c = 0.004 ^M^** **b–c = 0.012 ^M^**
**PLT**	303.7 ± 57.7	300.5 (256.0–354.0)	310.9 ± 91.4	287.0 (244.0–368.0)	311.3 ± 87.4	296.0 (241.5–358.0)	0.855 ^A^
**RDW**	13.3 ± 1.4	13.0 (12.3–13.7)	14.0 ± 0.9	12.9 (12.4–13.7)	13.1 ± 1.0	12.9 (12.5–13.3)	0.607 ^A^
**RDW/PLT**	0.05± 0.01	0.0 (0.0–0.1)	0.05 ± 0.01	0.0 (0.0–0.1)	0.05 ± 0.01	0.0 (0.0–0.1)	0.970 ^A^
**PLT/WBC**	28.3 ± 10.5	26.1 (21.0–35.1)	27.6 ± 12.2	23.3 (18.8–34.6)	22.1 ± 8.7	20.1 (17.1–25.3)	**0.014 ^A^** **a–c = 0.004 ^M^**
**Na**	137.4 ± 2.1	137.5 (136.0–139.0)	137.6 ± 2.3	138.0 (136.0–139.0)	136.1 ± 2.7	136.0 (135.0–138.0)	**0.008 ^A^** **b–c = 0.012 ^M^**
**CRP**	27.1 ± 52.3	5.2 (1.2–19.1)	26.7 ± 47.0	8.3 (2.6–25.8)	77.2 ± 71.7	61.2 (11.6–134.5)	**<0.001 ^K^** **a–c < 0.001 ^M^** **b–c < 0.001 ^M^**
**Procalcitonin**	0.4 ± 2.3	0.1 (0.0–0.2)	0.2 ± 0.6	0.0 (0.0–0.1)	0.8 ± 1.7	0.1 (0.0–0.5)	**0.011** **^K^** **b** **–** **c = 0.003** **^M^**
**Fibrinogen**	323.6 ± 66.0	319.5 (276.0–363.0)	444.2 ± 63.5	435.0 (407.0–491.0)	541.6 ± 59.5	526.0 (495.5–567.5)	**<0.001 ^A^** **a–b < 0.001 ^M^** **a–c < 0.001 ^M^** **b–c < 0.001 ^M^**
**AST**	25.7 ± 9.0	23.5 (20.0–30.0)	23.1 ± 9.4	21.5 (18.5–26.5)	22.2 ± 5.2	22.0 (18.5–25.5)	0.071 ^K^
**ALT**	15.7 ± 12.5	13.5 (10.0–18.0)	14.7 ± 9.5	13.0 (10.0–15.0)	13.3 ± 4.6	12.0 (10.0–15.5)	0.932 ^K^
**Albumin**	4.6 ± 0.3	4.7 (4.4–4.8)	4.2 ± 0.4	4.1 (3.9–4.3)	4.3 ± 0.5	4.3 (4.0–4.5)	**<0.001 ^A^** **a–b < 0.001 ^M^** **a–c < 0.001 ^M^**

^A^, One-Way ANOVA test; ^K^, Kruskal–Wallis test; *, Ki-kare test; ^M^, The Bonferroni-corrected Mann–Whitney U test was applied; 0.05/3 ≈ *p* < 0.017 was considered significant. Abbreviations: ALS, absolute lymphocyte count; ALT, alanine aminotransferase; ANC, absolute neutrophil count; AST, aspartate aminotransferase; CRP, C-reactive protein; MLR, monocyte-to-lymphocyte ratio; Na, sodium; NLR, neutrophil-to-lymphocyte ratio; PLT, platelet count; PLT/WBC, platelet-to-white blood cell ratio; RDW, red cell distribution width; RDW/PLT, red cell distribution width-to-platelet ratio; WBC, white blood cell count.

**Table 2:** Comparison of demographic and biochemical parameters between appendicitis and healthy/NSAP groups.

Variable		Healthy Controls (n = 62)		Appendicitis (n = 83)		*p*
**Sex**	**Female, n (%)**	36 (58.1)		30 (36.1)		**0.009 ^K^**
	male, n (%)	26 (41.9)		53 (63.9)		
		**Mean ± SD**	**Median** **(Q1** **–** **Q3)**	**Mean ± SD**	**Median** **(Q1** **–** **Q3)**	
Age (month)		156.35 ± 41.98	165.5 (126.0–189.0)	153.72 ± 45.22	160.0 (122.0–196.0)	0.831 ^M^
WBC		12.1 ± 4.6	11.2 (8.6–14.9)	13.8 ± 5.1	13.3 (10.6–16.5)	**0.033 ^T^**
ANS		8.8 ± 4.7	7.8 (5.0–11.6)	10.7 ± 5.0	9.7 (7.3–13.9)	**0.012 ^M^**
ALS		2.3 ± 1.4	2.2 (1.2–3.0)	2.0 ± 0.9	1.9 (1.3–2.5)	0.310 ^M^
NLR		6.3 ± 6.4	3.5 (1.9–7.3)	6.7 ± 5.0	5.4 (3.4–8.6)	**0.028 ^M^**
Monocyte		0.8 ± 0.4	0.7 (0.5–1.1)	0.9 ± 0.4	0.8 (0.7–1.2)	**0.015 ^M^**
MLR		0.5 ± 0.3	0.4 (0.2–0.6)	0.6 ± 0.4	0.5 (0.3–0.7)	0.111 ^M^
PLT		303.7 ± 57.7	300.5 (256.0–354.0)	311.1 ± 89.7	291.0 (242.0–366.5)	0.552 ^T^
RDW		13.3 ± 1.4	13.0 (12.3–13.7)	13.1 ± 1.0	12.9 (12.5–13.5)	0.324 ^T^
RDW/PLT		0.0 ± 0.0	0.0 (0.0–0.1)	0.0 ± 0.0	0.0 (0.0–0.1)	0.950 ^M^
PLT/WBC		28.3 ± 10.5	26.1 (21.0–35.1)	25.1 ± 11.0	21.6 (17.6–29.1)	**0.015 ^M^**
Na		137.4 ± 2.1	137.5 (136.0–139.0)	136.9 ± 2.6	137.0 (136.0–138.0)	0.222 ^T^
CRP		27.1 ± 52.3	5.2 (1.2–19.1)	50.4 ± 64.7	16.2 (6.1–93.6)	**<0.001 ^M^**
Procalcitonin		0.4 ± 2.3	0.1 (0.0–0.2)	0.4 ± 1.3	0.1 (0.0–0.1)	0.848 ^M^
Fibrinogen		323.6 ± 66.0	319.5 (276.0–363.0)	489.9 ± 78.4	491.0 (426.5–530.0)	**<0.001 ^T^**
AST		25.7 ± 9.0	23.5 (20.0–30.0)	22.7 ± 7.7	22.0 (18.5–26.0)	**0.021 ^M^**
ALT		15.7 ± 12.5	13.5 (10.0–18.0)	14.1 ± 7.6	13.0 (10.0–15.5)	0.708 ^M^
Albumin		4.6 ± 0.3	4.7 (4.4–4.8)	4.2 ± 0.4	4.1 (3.9–4.5)	**<0.001 ^T^**

^T^; Student T testi, ^M^; Mann–Whitney U testi, ^K^; Ki-kare testi. Abbreviations: ALS, absolute lymphocyte count; ALT, alanine aminotransferase; ANC, absolute neutrophil count; AST, aspartate aminotransferase; CRP, C-reactive protein; MLR, monocyte-to-lymphocyte ratio; Na, sodium; NLR, neutrophil-to-lymphocyte ratio; PLT, platelet count; PLT/WBC, platelet-to-white blood cell ratio; RDW, red cell distribution width; RDW/PLT, red cell distribution width-to-platelet ratio; WBC, white blood cell count.

**Table 3:** Distribution of variables among patients with perforated, gangrenous, and peritonitic appendicitis.

Variable	Perforated (n = 15)		Gangrenous (n = 14)		Peritonitis (n = 10)		*p*
**Sex**							
Female, n (%)	1 (6.7)		3 (21.4)		3 (30.0)		0.302 *
Male, n (%)	14 (93.3)		11 (78.6)		7 (70.0)		
	**Mean ± SD**	**Median** **(Q1** **–** **Q3)**	**Mean ± SD**	**Median** **(Q1** **–** **Q3)**	**Mean ± SD**	**Median** **(Q1** **–** **Q3)**	
**Age (month)**	143.5 ± 59.6	168.0 (86.0–196.0)	161.1 ± 31.9	160.5 (131.0–190.0)	158.7 ± 30.3	162.0 (126.0–175.0)	0.528 ^K^
**WBC**	15.1 ± 6.0	14.8 (10.6–17.3)	14.8 ± 4.4	14.8 (11.5–17.1)	15.6 ± 3.9	16.7 (12.9–18.4)	0.916 ^A^
**ANS**	11.9 ± 5.8	11.0 (8.2–15.1)	11.8 ± 4.3	12.1 (8.8–13.9)	12.4 ± 3.5	13.5 (10.0–14.1)	0.946 ^A^
**ALS**	1.8 ± 1.2	1.5 (0.9–2.4)	1.8 ± 0.6	1.8 (1.4–2.1)	2.0 ± 0.6	1.9 (1.5–2.6)	0.722 ^A^
**NLR**	9.0 ± 6.4	8.5 (5.1–10.0)	8.3 ± 6.9	6.4 (4.2–10.4)	6.5 ± 2.5	5.7 (4.8–8.9)	0.586 ^K^
**Monocyte**	0.9 ± 0.5	0.8 (0.7–1.1)	1.1 ± 0.4	1.2 (0.8–1.6)	1.1 ± 0.4	1.1 (0.8–1.3)	0.449 ^K^
**MLR**	0.6 ± 0.4	0.5 (0.4–0.8)	0.8 ± 0.6	0.6 (0.4–1.0)	0.5 ± 0.2	0.5 (0.4–0.6)	0.401 ^K^
**PLT**	289.9 ± 66.5	281.0 (246.5–328.0)	319.4 ± 111.3	296.0 (235.0–372.0)	332.0 ± 84.9	320.0 (261.0–379.0)	0.478 ^K^
**RDW**	13.2 ± 0.8	13.1 (12.7–13.3)	12.9 ± 1.0	12.8 (12.4–13.2)	13.1 ± 1.3	13.1 (12.1–13.8)	0.728 ^A^
**RDW/PLT**	0.04± 0.01	0.1 (0.0–0.1)	0.04 ± 0.01	0.0 (0.0–0.1)	0.04 ± 0.01	0.0 (0.0–0.1)	0.403 ^A^
**PLT/WBC**	21.1 ± 7.6	16.4 (19.0–24.3)	23.5 ± 11.7	18.1 (19.9–25.7)	21.8 ± 4.9	18.6 (21.4–23.6)	0.762 ^K^
**Na**	135.0 ± 2.8	134.0 (136.0–136.0)	136.6 ± 2.1	136.0 (136.0–138.0)	137.0 ± 3.1	136.0 (137.0–140.0)	0.132 ^A^
**CRP**	96.5 ± 85.1	15.6 (97.1–137.7)	80.6 ± 63.1	13.7 (89.5–137.1)	43.4 ± 52.5	7.7 (19.8–74.9)	0.191 ^K^
**Procalcitonin**	1.0 ± 1.5	0.0 (0.1–1.4)	0.9 ± 2.4	0.0 (0.1–0.5)	0.2 ± 0.3	0.0 (0.1–0.3)	0.498 ^K^
**Fibrinogen**	558.1 ± 71.0	511.0 (534.0–583.0)	546.6 ± 50.5	499.0 (557.5–568.0)	509.8 ± 42.3	483.0 (496.5–513.0)	0.128 ^A^
**AST**	23.5 ± 5.6	19.0 (24.0–26.0)	21.1 ± 4.6	19.0 (22.0–24.0)	21.6 ± 5.6	18.0 (20.5–22.0)	0.464 ^A^
**ALT**	13.3 ± 4.6	10.5 (12.0–15.5)	12.4 ± 3.8	10.0 (11.5–16.0)	13.8 ± 5.8	10.0 (13.5–15.0)	0.666 ^A^
**Albumin**	4.1 ± 0.4	4.0 (4.1–4.3)	4.3 ± 0.6	4.0 (4.2–4.5)	4.5 ± 0.5	4.3 (4.5–4.9)	0.324 ^A^

^A^; One-Way ANOVA testi, ^K^; Kruskal–Wallis testi, *; Ki-kare testi. Abbreviations: ALS, absolute lymphocyte count; ALT, alanine aminotransferase; ANC, absolute neutrophil count; AST, aspartate aminotransferase; CRP, C-reactive protein; MLR, monocyte-to-lymphocyte ratio; Na, sodium; NLR, neutrophil-to-lymphocyte ratio; PLT, platelet count; PLT/WBC, platelet-to-white blood cell ratio; RDW, red cell distribution width; RDW/PLT, red cell distribution width-to-platelet ratio; WBC, white blood cell count.

**Table 4:** Results of ROC analysis (appendicitis vs. control group).

Variable	AUC (%95 Cl)	Cut-Off	Sensitivity	Specificity	Youden	*p*	PPV	NPV
**WBC**	0.61 (0.52–0.71)	11.6	65.1%	%56.5	0.215	0.019	%66.7	%90.3
**ANS**	0.62 (0.53–0.72)	7.0	%78.3	%48.4	0.267	0.012	%66.7	%85.4
**NLR**	0.61 (0.51–0.71)	3.1	%81.9	%45.2	0.271	0.028	%67.0	%62.5
**Monocyte**	0.62 (0.53–0.71)	0.6	%84.3	%41.9	0.263	0.015	%65.7	%65.0
**PLT/WBC**	0.38 (0.29–0.47)	-	-	-	0.003	0.015		
**CRP**	0.68 (0.59–0.77)	6.4	%74.7	%59.7	0.344	<0.001	%64.2	%63.2
**Fibrinogen**	0.95 (0.92–0.98)	395.0	%92.8	%90.3	0.831	<0.001	%92.8	%90.3
**AST**	0.39 (0.29–0.48)	-	-	-	0.016	0.022		
**Albumin**	0.24 (0.16–0.32)	-	-	-	0.052	<0.001		

Abbreviations: AUC, area under the curve; ANS, absolute neutrophil count; NLR, neutrophil-to-lymphocyte ratio; PLT/WBC, platelet-to-white blood cell ratio; CRP, C-reactive protein; PPV, positive predictive value; NPV, negative predictive value. Cut-off values were determined according to the maximum Youden index obtained from ROC curve analysis.

**Table 5:** Multivariate logistic regression analysis of factors associated with appendicitis.

Variable	B	S.E.	*p* Value	Exp(B) (OR)	95% CI for Exp(B)
Gender (Male)	0.750	0.756	0.321	2.117	0.481–9.311
WBC	−0.055	0.137	0.686	0.946	0.724–1.237
NLR	−0.133	0.084	0.111	0.875	0.743–1.031
Monocyte	−0.480	1.750	0.784	0.619	0.020–19.103
PLT/WBC	−0.055	0.049	0.258	0.946	0.860–1.041
**CRP**	−0.031	0.013	**0.016**	0.969	0.945–0.994
**Fibrinogen**	0.047	0.010	**<0.001**	1.048	1.028–1.068
**AST**	−0.083	0.041	**0.045**	0.921	0.849–0.998
**Albumin**	−3.119	1.096	**0.004**	0.044	0.005–0.379

Abbreviations: AST, aspartate aminotransferase; CRP, C-reactive protein; NLR, neutrophil-to-lymphocyte ratio; PLT/WBC, platelet-to-white blood cell ratio; WBC, white blood cell count.

## Data Availability

The data presented in this study are available from the corresponding author upon reasonable request. The data are not publicly available due to privacy and ethical restrictions related to patient information.

## References

[1] Kliegman R.M., St. Geme J.W., Blum N.J., Shah S.S., Tasker R.C., Wilson K.M. (2020). Abdominal Pain in Children. Nelson Textbook of Pediatrics.

[2] Çelik B., Nalçacıoğlu H., Özçatal M., Altuner Torun Y. (2019). Role of neutrophil-to-lymphocyte ratio and platelet-to-lymphocyte ratio in identifying complicated appendicitis in the pediatric emergency department. Ulus. Travma Acil Cerrahi Derg..

[3] El-Sayed M.A., Hassan A.M., Abdel-Aziz M., Mohamed H.F. (2022). Role of Procalcitonin as a Predictor of Complicated Acute Appendicitis. Med. J. Cairo Univ..

[4] Prada Arias M., Salgado Barreira A., Montero Sánchez M., Fernández Eire P., García Saavedra S., Gómez Veiras J., Fernández Lorenzo J.R. (2018). Appendicitis versus non-specific acute abdominal pain: Paediatric Appendicitis Score evaluation. An. Pediatr..

[5] Gongidi P., Bellah R.D. (2017). Ultrasound of the Pediatric Appendix. Pediatr. Radiol..

[6] Russell W.S., Schuh A.M., Hill J.G., Hebra A., Cina R.A., Smith C.D., Streck C.J. (2013). Clinical practice guidelines for pediatric appendicitis evaluation can decrease computed tomography utilization while maintaining diagnostic accuracy. Pediatr. Emerg. Care.

[7] Zhou J., Xu W., Wang J., Fan Z. (2022). Relevant markers used in identifying complicated appendicitis in children. Front. Pharmacol..

[8] Hernández-González L.L., Serrano-Guzmán J., Guzmán-Ortiz J.D., Pérez-Ceballos H.E., Cano-Pérez J.L., Cruz-Hernández V., Bernardino-Hernández H.U., Martínez-Martínez L.L., Aguilar-Ruiz S.R. (2025). C-Reactive protein, international normalized ratio, and fibrinogen in the diagnostic scale of complicated acute appendicitis. Clin. Pract..

[9] Alvarez-Alvarez F.A., Maciel-Gutierrez V.M., Rocha-Muñoz A.D., Lujan J.H., Ploneda-Valencia C.F. (2016). Diagnostic value of serum fibrinogen as a predictive factor for complicated (perforated) appendicitis: A cross-sectional study. Int. J. Surg..

[10] Vinod Kumar M.S., Tiwari M.K., Singh J., Malik A. (2021). Plasma fibrinogen: An independent predictor of appendicitis in children. J. Indian Assoc. Pediatr. Surg..

[11] Wu Z., Zhao L., Liu Y., Qian S., Wu L., Liu X. (2022). Fibrinogen as a Marker of Overall and Complicated Acute Appendicitis: A Systematic Review and Meta-Analysis. J. Surg. Res..

[12] Eun S., Ho I.G., Bae G.E., Kim H., Koo C.M., Kim M.K., Yoon S.H. (2021). Neutrophil-to-lymphocyte ratio in the diagnosis of acute appendicitis in children: A systematic review and meta-analysis. Eur. Rev. Med. Pharmacol. Sci..

[13] Ding X., Li Y., Yu D., Huang Q., Wang S.M., Bai J., Pan Y.B., Mahamat D.A., Yang L., Wu K. (2025). Predictive value of neutrophil, C-reactive protein, fibrinogen, and chloride for acute complicated appendicitis in children: A multicenter retrospective study. Pediatr. Surg. Int..

[14] Podda M., Ceresoli M., De Simone B., Fugazzola P., Pata F., Balla A., Gerardi C., Allocati E., Salminen P., Coimbra R. (2026). Diagnosis and treatment of acute appendicitis: 2025 update of the WSES Jerusalem guidelines. JAMA Surg..

[15] Kumar S.S., Collings A.T., Lamm R., Haskins I.N., Scholz S., Nepal P., Train A.T., Athanasiadis D.I., Pucher P.H., Bradley J.F. (2024). SAGES guideline for the diagnosis and treatment of appendicitis. Surg. Endosc..

[16] Gorter R.R., Eker H.H., Gorter-Stam M.A.W., Abis G.S.A., Acharya A., Ankersmit M., Antoniou S.A., Arolfo S., Babic B., Boni L. (2016). Diagnosis and management of acute appendicitis: EAES consensus development conference 2015. Surg. Endosc..

[17] Prada-Arias M., Vázquez J.L., Salgado-Barreira Á., Gómez-Veiras J., Montero-Sánchez M., Fernández-Lorenzo J.R. (2017). Diagnostic accuracy of fibrinogen in differentiating appendicitis from nonspecific abdominal pain in children. Am. J. Emerg. Med..

[18] Narsat M.A., Gülten S., Yıldız E., Durak Ö., Yılmaz A. (2022). Comparison of inflammatory markers in the differentiation of uncomplicated appendicitis and complicated appendicitis. Kastamonu Med. J..

[19] Nyuwi K.T., Singh C.G., Khumukcham S., Rangaswamy R., Ezung Y.S., Chittvolu S.R., Sharma A.B., Singh H.M. (2017). The Role of Serum Fibrinogen Level in the Diagnosis of Acute Appendicitis. J. Clin. Diagn. Res..

[20] Shafagh S., Barooni M., Davoodabadi A., Gilasi H., Hajian A. (2022). Evaluation of plasma level of fibrinogen as a diagnostic criterion in acute appendicitis; Cohort study. Ann. Med. Surg..

